# (Benzyl isocyanide-κ*C* ^1^)chlorido(2-chloro-3-dimethyl­amino-1-phenyl­prop-1-en-1-yl-κ^2^ *C* ^1^,*N*)palladium(II)

**DOI:** 10.1107/S1600536812049768

**Published:** 2012-12-08

**Authors:** Ana C. Mafud, Milene A. R. Oliviera, Maria T. P. Gambardella

**Affiliations:** a Instituto de Fisica de Sao Carlos, Av. do Trabalhador Saocarlense, 400, Sao Carlos, SP, Brazil; b Instituto de Quimica de Sao Carlos, Av. do Trabalhador Saocarlense, 400, Sao Carlos, SP, Brazil

## Abstract

In the title compound, [Pd(C_11_H_13_ClN)Cl(C_8_H_7_N)], which crystallized in the chiral space group *P*2_1_2_1_2_1_, the Pd^II^ atom is coordinated by two C atoms, a C*sp* ^2^ atom of the 2-chloro-3-dimethyl­amino-1-phenyl­prop-1-en-1-yl ligand and a C*sp* atom from the benzyl isocyanide ligand, as well as an N atom of the ligand and a Cl atom, in a square-planar geometry. In the complex, there is a short C—H⋯Cl hydrogen bond and a C—H⋯π inter­action. In the crystal, mol­ecules are linked *via* C—H⋯Cl hydrogen bonds, forming chains along the *a-*axis direction.

## Related literature

For the crystal structures of similar compounds, see: Moro *et al.* (2004 ▶); Caires *et al.* (2006 ▶); Mafud *et al.* (2013 ▶). 
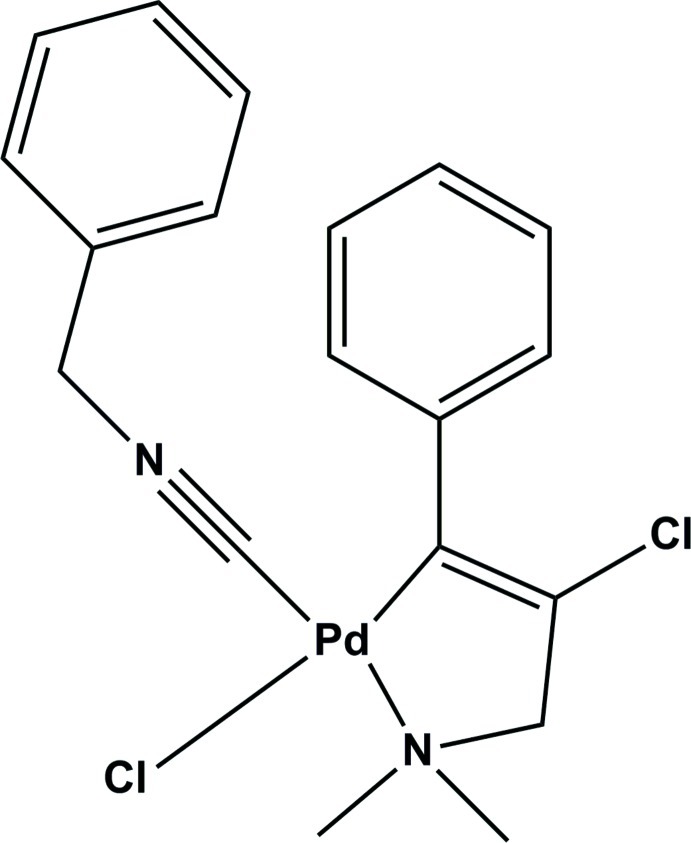


## Experimental

### 

#### Crystal data

[Pd(C_11_H_13_ClN)Cl(C_8_H_7_N)]
*M* *_r_* = 453.67
Orthorhombic, 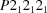

*a* = 6.2529 (7) Å
*b* = 11.0931 (10) Å
*c* = 27.640 (2) Å
*V* = 1917.2 (3) Å^3^
*Z* = 4
Mo *K*α radiation
μ = 1.25 mm^−1^
*T* = 290 K
0.63 × 0.08 × 0.05 mm

#### Data collection

Enraf–Nonius TurboCAD-4 diffractometer
Absorption correction: ψ scan (North *et al.*, 1968 ▶) *T*_min_ = 0.871, *T*_max_ = 0.928
3421 measured reflections
3358 independent reflections
2420 reflections with *I* > 2σ(*I*)
*R*_int_ = 0.019
3 standard reflections every 120 min intensity decay: 1%

#### Refinement

*R*[*F* ^2^ > 2σ(*F* ^2^)] = 0.039
*wR*(*F* ^2^) = 0.085
*S* = 1.03
3358 reflections
219 parameters
H-atom parameters constrained
Δρ_max_ = 0.67 e Å^−3^
Δρ_min_ = −1.07 e Å^−3^
Absolute structure: Flack (1983 ▶), 161 Friedel pairs
Flack parameter: −0.10 (5)

### 

Data collection: *CAD-4 EXPRESS* (Enraf–Nonius, 1994 ▶); cell refinement: *CAD-4 EXPRESS*; data reduction: *XCAD4* (Harms & Wocadlo, 1995 ▶); program(s) used to solve structure: *SHELXS97* (Sheldrick, 2008 ▶); program(s) used to refine structure: *SHELXL97* (Sheldrick, 2008 ▶); molecular graphics: *ORTEP-3 for Windows* (Farrugia, 2012 ▶) and *Mercury* (Macrae *et al.*, 2008 ▶); software used to prepare material for publication: *WinGX* (Farrugia, 2012 ▶).

## Supplementary Material

Crystal structure: contains datablock(s) I, global. DOI: 10.1107/S1600536812049768/su2529sup1.cif

Structure factors: contains datablock(s) I. DOI: 10.1107/S1600536812049768/su2529Isup2.hkl

Additional supplementary materials:  crystallographic information; 3D view; checkCIF report

## Figures and Tables

**Table 1:** Hydrogen-bond geometry (Å, °) *Cg*1 is the centroid of the C10–C15 ring.

*D*—H⋯*A*	*D*—H	H⋯*A*	*D*⋯*A*	*D*—H⋯*A*
C19—H19*B*⋯Cl1	0.96	2.65	3.275 (8)	123
C17—H17*A*⋯Cl1^i^	0.97	2.80	3.729 (6)	160
C4—H4⋯*Cg*1	0.93	2.76	3.622 (8)	155

Symmetry code: (i) 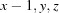.

**Fig. 1.**
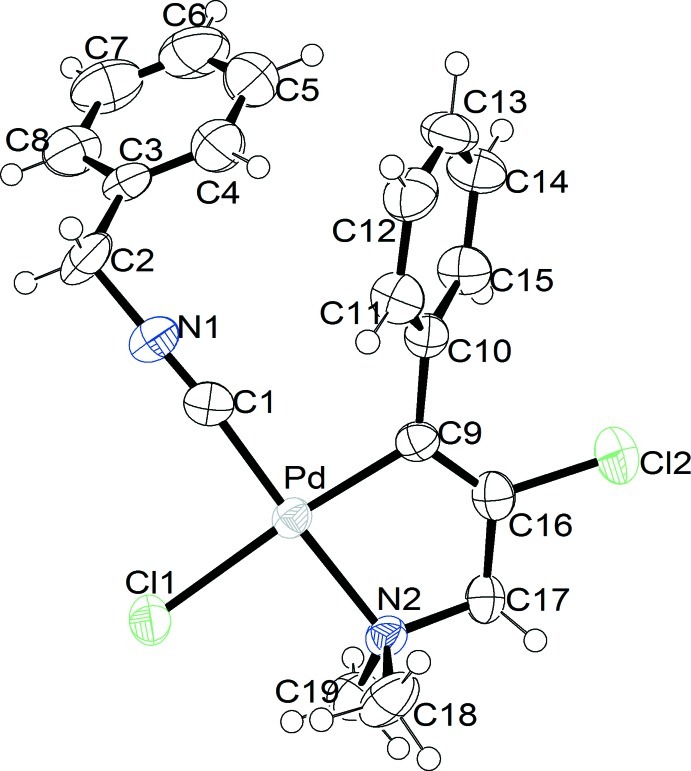
Perspective view of the molecular structure of the title compound, with the atom numbering. Displacement ellipsoids are drawn at the 50% probability level.

**Fig. 2.**
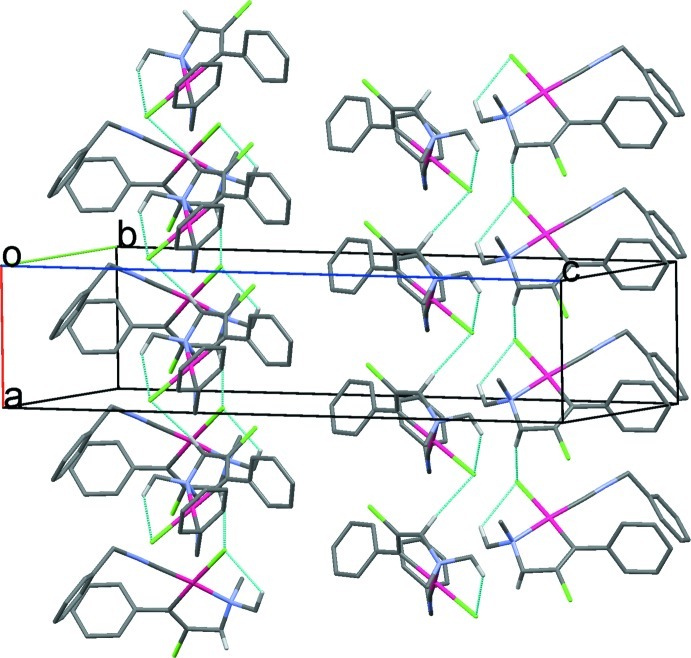
The view along the b axis of the crystal packing of the title compound. The C-H···Cl interactions are shown as dashed cyan lines and the H atoms not involved in these interactions have been omitted for clarity.

## supplementary crystallographic information

### Comment

The title compound was obtained from the reaction between the dimer [Pd(DMBA)(µ*X*)]_2_ [where *X* = Cl, N_3_, NCO, and DMBA = 7,12-dimethylbenz(a)anthracene] and thiourea, being the product of a cleavage reaction. As a palladium complex it could be of interest with respect to anticancer activity.

In the title compound, Fig. 1, the palladium atom coordinates to two C atoms, a C_sp2_ and a C_sp_ atom [Pd1—C9 2.006 (5) Å, Pd1—C1 1.928 (6) Å, respectively], the amine N atom [Pd1—N2 2.098 (4) Å] and an atom of chlorine [Pd1—Cl1 2.3929 (2) Å], with a square planar geometry. The distances and angles in the title compound are close to those reported for similar compounds (Moro *et al.*, 2004; Caires *et al.*, 2006; Mafud *et al.*, 2013). In the molecule there is a short C-H···Cl contact and a C-H···π interaction (Table 1).

In the crystal, molecules are linked via C-H···Cl hydrogen bonds (Fig. 2 and Table 1) forming chains along the a axis.

### Experimental

The title compound is the product of a cleavage reaction. It was obtained from the reaction between the dimer [Pd (DMBA)(µ*X*)]_2_ [where *X* = Cl, N_3_, NCO, and DMBA = 7,12-dimethylbenz(a)anthracene] and thiourea, in a 1:2 stoichiometric ratio in chloroform. The solution was stirred during 1 h and then the mixture was left for the solvent to slowly evaporate at room temperature. Large yellow needle-shaped crystals, suitable for X-ray diffraction analysis, were obtained.

### Refinement

The C-bound H-atoms were included in calculated positions and treated as riding atoms: C—H = 0.93, 0.96 and 0.97 Å, for CH, CH_3_ and CH_2_ H atoms, respectively, with *U*_iso_(H) = k × *U*_eq_(parent C-atom), were k = 1.5 for CH_3_ H atoms, and = 1.2 for other H atoms.

### Crystal data

**Table:**

[Pd(C_11_H_13_ClN)Cl(C_8_H_7_N)]	*F*(000) = 912
*M**_r_* = 453.67	*D*_x_ = 1.572 Mg m^−^^3^
Orthorhombic, *P*2_1_2_1_2_1_	Mo *K*α radiation, λ = 0.71073 Å
Hall symbol: P 2ac 2ab	Cell parameters from 25 reflections
*a* = 6.2529 (7) Å	θ = 11.2–18.2°
*b* = 11.0931 (10) Å	µ = 1.25 mm^−^^1^
*c* = 27.640 (2) Å	*T* = 290 K
*V* = 1917.2 (3) Å^3^	Prism, yellow
*Z* = 4	0.63 × 0.08 × 0.05 mm

### Data collection

**Table:**

Enraf–Nonius TurboCAD-4 diffractometer	2420 reflections with *I* > 2σ(*I*)
Radiation source: Enraf Nonius FR590	*R*_int_ = 0.019
Graphite monochromator	θ_max_ = 29.9°, θ_min_ = 2.9°
non–profiled ω scans	*h* = −8→1
Absorption correction: ψ scan (North *et al.*, 1968)	*k* = −15→0
*T*_min_ = 0.871, *T*_max_ = 0.928	*l* = 0→38
3421 measured reflections	3 standard reflections every 120 min
3358 independent reflections	intensity decay: 1%

### Refinement

**Table:**

Refinement on *F*^2^	Secondary atom site location: difference Fourier map
Least-squares matrix: full	Hydrogen site location: inferred from neighbouring sites
*R*[*F*^2^ > 2σ(*F*^2^)] = 0.039	H-atom parameters constrained
*wR*(*F*^2^) = 0.085	*w* = 1/[σ^2^(*F*_o_^2^) + (0.0337*P*)^2^] where *P* = (*F*_o_^2^ + 2*F*_c_^2^)/3
*S* = 1.03	(Δ/σ)_max_ = 0.001
3358 reflections	Δρ_max_ = 0.67 e Å^−^^3^
219 parameters	Δρ_min_ = −1.07 e Å^−^^3^
0 restraints	Absolute structure: Flack (1983), 161 Friedel pairs
Primary atom site location: structure-invariant direct methods	Flack parameter: −0.10 (5)

### Special details

**Table:**

Geometry. All e.s.d.'s (except the e.s.d. in the dihedral angle between two l.s. planes) are estimated using the full covariance matrix. The cell e.s.d.'s are taken into account individually in the estimation of e.s.d.'s in distances, angles and torsion angles; correlations between e.s.d.'s in cell parameters are only used when they are defined by crystal symmetry. An approximate (isotropic) treatment of cell e.s.d.'s is used for estimating e.s.d.'s involving l.s. planes.
Refinement. Refinement of *F*^2^ against ALL reflections. The weighted *R*-factor *wR* and goodness of fit *S* are based on *F*^2^, conventional *R*-factors *R* are based on *F*, with *F* set to zero for negative *F*^2^. The threshold expression of *F*^2^ > σ(*F*^2^) is used only for calculating *R*-factors(gt) *etc*. and is not relevant to the choice of reflections for refinement. *R*-factors based on *F*^2^ are statistically about twice as large as those based on *F*, and *R*- factors based on ALL data will be even larger.

### Fractional atomic coordinates and isotropic or equivalent isotropic displacement parameters (Å^2^)

**Table:**

	*x*	*y*	*z*	*U*_iso_*/*U*_eq_	
Pd1	0.69687 (7)	0.16519 (4)	0.302107 (14)	0.03072 (10)	
Cl2	0.1519 (3)	0.35671 (17)	0.37641 (6)	0.0620 (5)	
Cl1	0.9388 (3)	0.12673 (13)	0.23683 (5)	0.0460 (4)	
N1	0.8931 (9)	−0.0511 (5)	0.35548 (17)	0.0432 (12)	
N2	0.5455 (8)	0.3051 (4)	0.26417 (15)	0.0336 (11)	
C1	0.8195 (11)	0.0332 (5)	0.33801 (18)	0.0374 (13)	
C2	0.9749 (11)	−0.1613 (6)	0.3763 (2)	0.0584 (18)	
H2A	1.0798	−0.1418	0.4009	0.07*	
H2B	1.0462	−0.2078	0.3513	0.07*	
C3	0.7989 (12)	−0.2368 (5)	0.39854 (19)	0.0420 (13)	
C4	0.6530 (14)	−0.1880 (7)	0.4296 (2)	0.062 (2)	
H4	0.6605	−0.1065	0.4373	0.075*	
C5	0.4955 (16)	−0.2581 (9)	0.4496 (3)	0.084 (3)	
H5	0.3979	−0.2247	0.4712	0.1*	
C6	0.4832 (18)	−0.3770 (9)	0.4377 (3)	0.087 (3)	
H6	0.3748	−0.4244	0.4507	0.104*	
C7	0.6277 (17)	−0.4276 (7)	0.4068 (3)	0.082 (3)	
H7	0.6188	−0.509	0.3991	0.098*	
C8	0.7869 (14)	−0.3574 (6)	0.3870 (2)	0.0608 (19)	
H8	0.886	−0.3913	0.366	0.073*	
C9	0.4927 (9)	0.2110 (5)	0.35497 (18)	0.0325 (13)	
C10	0.4966 (9)	0.1640 (6)	0.40531 (18)	0.0364 (12)	
C11	0.6694 (11)	0.1801 (6)	0.43592 (19)	0.0458 (15)	
H11	0.7911	0.2193	0.4247	0.055*	
C12	0.6627 (11)	0.1380 (6)	0.4833 (2)	0.0514 (17)	
H12	0.7784	0.1514	0.5037	0.062*	
C13	0.4896 (14)	0.0777 (6)	0.4999 (2)	0.0551 (19)	
H13	0.4885	0.0486	0.5314	0.066*	
C14	0.3175 (13)	0.0594 (6)	0.4709 (2)	0.0538 (17)	
H14	0.198	0.0186	0.4825	0.065*	
C15	0.3225 (12)	0.1027 (5)	0.4235 (2)	0.0463 (15)	
H15	0.2048	0.0898	0.4036	0.056*	
C16	0.3523 (9)	0.2918 (5)	0.3399 (2)	0.0388 (14)	
C17	0.3425 (9)	0.3377 (6)	0.28883 (18)	0.0449 (14)	
H17A	0.2222	0.3018	0.272	0.054*	
H17B	0.3242	0.4246	0.2888	0.054*	
C18	0.6951 (13)	0.4088 (5)	0.2648 (2)	0.0585 (18)	
H18A	0.6372	0.4732	0.2457	0.088*	
H18B	0.8305	0.3845	0.2517	0.088*	
H18C	0.7144	0.4359	0.2975	0.088*	
C19	0.4974 (12)	0.2744 (6)	0.21329 (19)	0.0564 (19)	
H19A	0.4008	0.2072	0.2123	0.085*	
H19B	0.6275	0.2535	0.1969	0.085*	
H19C	0.4327	0.3425	0.1976	0.085*	

### Atomic displacement parameters (Å^2^)

**Table:**

	*U*^11^	*U*^22^	*U*^33^	*U*^12^	*U*^13^	*U*^23^
Pd1	0.02745 (18)	0.02875 (17)	0.03597 (17)	−0.0004 (2)	−0.0021 (2)	0.00213 (19)
Cl2	0.0515 (11)	0.0667 (12)	0.0677 (10)	0.0150 (10)	0.0197 (9)	0.0015 (9)
Cl1	0.0411 (9)	0.0440 (8)	0.0529 (8)	0.0055 (7)	0.0090 (7)	−0.0006 (7)
N1	0.041 (3)	0.037 (3)	0.052 (3)	−0.001 (3)	−0.009 (3)	0.008 (2)
N2	0.028 (2)	0.033 (3)	0.039 (2)	0.001 (2)	−0.001 (2)	0.004 (2)
C1	0.038 (3)	0.037 (3)	0.037 (3)	−0.011 (3)	−0.005 (3)	0.000 (2)
C2	0.058 (4)	0.042 (3)	0.075 (4)	0.014 (4)	−0.010 (4)	0.017 (4)
C3	0.048 (4)	0.033 (3)	0.045 (3)	−0.001 (4)	−0.006 (4)	0.012 (2)
C4	0.074 (6)	0.049 (4)	0.064 (4)	0.004 (4)	0.012 (4)	0.002 (3)
C5	0.068 (6)	0.094 (7)	0.088 (6)	0.018 (6)	0.021 (5)	0.027 (6)
C6	0.083 (8)	0.073 (6)	0.104 (7)	−0.016 (6)	0.001 (6)	0.040 (5)
C7	0.109 (9)	0.047 (4)	0.089 (6)	−0.018 (5)	−0.017 (6)	0.015 (4)
C8	0.082 (5)	0.051 (4)	0.049 (3)	0.004 (5)	−0.011 (4)	−0.002 (3)
C9	0.029 (3)	0.031 (3)	0.037 (3)	−0.005 (2)	−0.001 (2)	0.002 (2)
C10	0.035 (3)	0.035 (3)	0.039 (3)	−0.006 (3)	0.000 (2)	0.001 (3)
C11	0.040 (4)	0.050 (4)	0.047 (3)	−0.008 (4)	−0.004 (3)	−0.001 (3)
C12	0.052 (4)	0.059 (4)	0.043 (3)	0.006 (4)	−0.014 (3)	−0.002 (3)
C13	0.077 (5)	0.054 (4)	0.034 (3)	−0.001 (4)	0.010 (4)	0.005 (3)
C14	0.053 (4)	0.057 (4)	0.052 (3)	−0.015 (4)	0.008 (4)	0.012 (3)
C15	0.037 (4)	0.053 (4)	0.048 (3)	−0.012 (4)	−0.001 (3)	0.004 (3)
C16	0.030 (3)	0.040 (3)	0.046 (3)	−0.003 (3)	0.002 (3)	−0.005 (3)
C17	0.034 (3)	0.048 (3)	0.053 (3)	0.011 (3)	0.004 (2)	0.003 (3)
C18	0.051 (4)	0.034 (3)	0.091 (5)	−0.006 (4)	−0.009 (5)	0.015 (3)
C19	0.056 (5)	0.068 (5)	0.044 (3)	0.006 (4)	−0.010 (3)	0.012 (3)

### Geometric parameters (Å, º)

**Table:**

Pd1—C1	1.928 (6)	C8—H8	0.93
Pd1—C9	2.006 (5)	C9—C16	1.322 (8)
Pd1—N2	2.099 (4)	C9—C10	1.486 (7)
Pd1—Cl1	2.3927 (15)	C10—C15	1.379 (8)
Cl2—C16	1.762 (6)	C10—C11	1.384 (8)
N1—C1	1.148 (7)	C11—C12	1.390 (8)
N1—C2	1.445 (8)	C11—H11	0.93
N2—C19	1.478 (7)	C12—C13	1.353 (9)
N2—C18	1.483 (8)	C12—H12	0.93
N2—C17	1.485 (7)	C13—C14	1.357 (10)
C2—C3	1.513 (9)	C13—H13	0.93
C2—H2A	0.97	C14—C15	1.396 (7)
C2—H2B	0.97	C14—H14	0.93
C3—C4	1.365 (9)	C15—H15	0.93
C3—C8	1.377 (8)	C16—C17	1.503 (8)
C4—C5	1.371 (11)	C17—H17A	0.97
C4—H4	0.93	C17—H17B	0.97
C5—C6	1.363 (11)	C18—H18A	0.96
C5—H5	0.93	C18—H18B	0.96
C6—C7	1.364 (12)	C18—H18C	0.96
C6—H6	0.93	C19—H19A	0.96
C7—C8	1.376 (11)	C19—H19B	0.96
C7—H7	0.93	C19—H19C	0.96
			
C1—Pd1—C9	94.0 (2)	C10—C9—Pd1	125.7 (4)
C1—Pd1—N2	176.6 (2)	C15—C10—C11	117.2 (5)
C9—Pd1—N2	83.7 (2)	C15—C10—C9	120.1 (5)
C1—Pd1—Cl1	90.08 (18)	C11—C10—C9	122.7 (5)
C9—Pd1—Cl1	175.44 (16)	C10—C11—C12	120.6 (6)
N2—Pd1—Cl1	92.31 (12)	C10—C11—H11	119.7
C1—N1—C2	176.5 (6)	C12—C11—H11	119.7
C19—N2—C18	108.6 (5)	C13—C12—C11	120.7 (6)
C19—N2—C17	108.6 (5)	C13—C12—H12	119.7
C18—N2—C17	110.1 (5)	C11—C12—H12	119.7
C19—N2—Pd1	113.4 (4)	C12—C13—C14	120.6 (6)
C18—N2—Pd1	106.5 (4)	C12—C13—H13	119.7
C17—N2—Pd1	109.7 (3)	C14—C13—H13	119.7
N1—C1—Pd1	173.8 (5)	C13—C14—C15	119.0 (7)
N1—C2—C3	111.9 (5)	C13—C14—H14	120.5
N1—C2—H2A	109.2	C15—C14—H14	120.5
C3—C2—H2A	109.2	C10—C15—C14	122.0 (7)
N1—C2—H2B	109.2	C10—C15—H15	119
C3—C2—H2B	109.2	C14—C15—H15	119
H2A—C2—H2B	107.9	C9—C16—C17	123.6 (5)
C4—C3—C8	119.6 (7)	C9—C16—Cl2	124.7 (5)
C4—C3—C2	121.5 (6)	C17—C16—Cl2	111.7 (4)
C8—C3—C2	118.9 (7)	N2—C17—C16	108.3 (5)
C3—C4—C5	120.6 (7)	N2—C17—H17A	110
C3—C4—H4	119.7	C16—C17—H17A	110
C5—C4—H4	119.7	N2—C17—H17B	110
C6—C5—C4	119.4 (9)	C16—C17—H17B	110
C6—C5—H5	120.3	H17A—C17—H17B	108.4
C4—C5—H5	120.3	N2—C18—H18A	109.5
C5—C6—C7	120.8 (9)	N2—C18—H18B	109.5
C5—C6—H6	119.6	H18A—C18—H18B	109.5
C7—C6—H6	119.6	N2—C18—H18C	109.5
C6—C7—C8	119.7 (8)	H18A—C18—H18C	109.5
C6—C7—H7	120.2	H18B—C18—H18C	109.5
C8—C7—H7	120.2	N2—C19—H19A	109.5
C7—C8—C3	119.8 (8)	N2—C19—H19B	109.5
C7—C8—H8	120.1	H19A—C19—H19B	109.5
C3—C8—H8	120.1	N2—C19—H19C	109.5
C16—C9—C10	122.9 (5)	H19A—C19—H19C	109.5
C16—C9—Pd1	111.4 (4)	H19B—C19—H19C	109.5
			
C1—Pd1—N2—C19	−90 (3)	Cl1—Pd1—C9—C16	35 (2)
C9—Pd1—N2—C19	−136.9 (4)	C1—Pd1—C9—C10	10.9 (5)
Cl1—Pd1—N2—C19	45.2 (4)	N2—Pd1—C9—C10	−171.6 (5)
C1—Pd1—N2—C18	151 (3)	Cl1—Pd1—C9—C10	−144.0 (18)
C9—Pd1—N2—C18	103.8 (4)	C16—C9—C10—C15	61.4 (8)
Cl1—Pd1—N2—C18	−74.1 (4)	Pd1—C9—C10—C15	−119.9 (6)
C1—Pd1—N2—C17	32 (3)	C16—C9—C10—C11	−117.9 (7)
C9—Pd1—N2—C17	−15.3 (4)	Pd1—C9—C10—C11	60.8 (8)
Cl1—Pd1—N2—C17	166.8 (3)	C15—C10—C11—C12	−1.5 (9)
C2—N1—C1—Pd1	−56 (14)	C9—C10—C11—C12	177.8 (6)
C9—Pd1—C1—N1	142 (5)	C10—C11—C12—C13	1.8 (10)
N2—Pd1—C1—N1	95 (6)	C11—C12—C13—C14	−1.3 (11)
Cl1—Pd1—C1—N1	−40 (5)	C12—C13—C14—C15	0.6 (11)
C1—N1—C2—C3	−42 (11)	C11—C10—C15—C14	0.8 (10)
N1—C2—C3—C4	−49.3 (9)	C9—C10—C15—C14	−178.6 (6)
N1—C2—C3—C8	130.8 (6)	C13—C14—C15—C10	−0.3 (10)
C8—C3—C4—C5	0.3 (11)	C10—C9—C16—C17	−178.3 (6)
C2—C3—C4—C5	−179.6 (7)	Pd1—C9—C16—C17	2.9 (7)
C3—C4—C5—C6	−1.0 (13)	C10—C9—C16—Cl2	0.5 (8)
C4—C5—C6—C7	1.2 (15)	Pd1—C9—C16—Cl2	−178.3 (3)
C5—C6—C7—C8	−0.7 (14)	C19—N2—C17—C16	143.6 (5)
C6—C7—C8—C3	−0.1 (12)	C18—N2—C17—C16	−97.6 (6)
C4—C3—C8—C7	0.2 (11)	Pd1—N2—C17—C16	19.2 (6)
C2—C3—C8—C7	−179.8 (6)	C9—C16—C17—N2	−15.7 (8)
C1—Pd1—C9—C16	−170.3 (4)	Cl2—C16—C17—N2	165.4 (4)
N2—Pd1—C9—C16	7.2 (4)		

### Hydrogen-bond geometry (Å, º)

Cg1 is the centroid of the C10–C15 ring.

**Table:**

*D*—H···*A*	*D*—H	H···*A*	*D*···*A*	*D*—H···*A*
C19—H19*B*···Cl1	0.96	2.65	3.275 (8)	123
C17—H17*A*···Cl1^i^	0.97	2.80	3.729 (6)	160
C4—H4···*Cg*1	0.93	2.76	3.622 (8)	155

Symmetry code: (i) *x*−1, *y*, *z*.
